# Mr. Gapper, on Digitalis

**Published:** 1802-02

**Authors:** Edm. Pitts Gapper

**Affiliations:** Ewell, Surrey


### *To the Editors of the Medical and Physical Journal.*

Gentlemen,

THE powerful effects of the Digitalis have so much engaged, of late, the attention of the medical part of the world, that we may confidently expect its real merits in the cure of diseases will ere long, by a liberal communication from those who employ it, be duly ascertained. Its power over the arterial system is so decided, as to lead to the rational conclusion, that by attentive, cautious, and judicious management, much good may be done with it; and we know, from the numerous communications which, through the medium of your excellent Journal, have been given to the world, that it may be administered with safety and effect. I have not been inattentive to its operations, and have been enabled by its use to afford very considerable relief to many patients in anasarcas and coughs, but, in no instance, with more clear and decided effect than in acute rheumatifm. It is a difeafe which, in the courfe of my practice, I have had numerous opportunities of feeing, and can bear ample teftimony to the difficulty in removing it, and to the length of time neceffary for that purpofe. I have principally relied on large and repeated bleedings early in the difeafe, and have ever found them effectual, joined with an antiphlogiftic regimen; but the debility which for the moft part fucceeds fuch a mode of treatment, is a matter of ferious confideration; and if a medicine can be difcovered which, by its effect on the arterial fyftem, will fuperfede the neceffity of bleeding, or even diminifh in any degree that neceffity, it will be a valuable difcovery; for it muft be allowed, that the lofs of fo large a quantity of blood as has frequently been found neceffary to remove the difeafe, muft have a very decided effect on the conftitution, and not unfrequently lays the foundation of irremediable mifchief. Having reflected much on thefe circumftances, and confidered the wonderful power which the Digitalis poffeffes of diminifhing the action of the heart and arteries, I was determined to employ it in the firft fully formed cafe I fhould meet with. Two cafes occurred nearly at the fame time, one only of which I fhall relate, as the treatment and event were the fame in both.

On the 15th of February, 1801, I was defired to vifit T. B. a robuft, healthy-looking man, in the prime of life, of a florid complexion and fanguine temperament. He had been feized on the 11th with rigors, fucceeded by fever, and very great pain. I found his hands, knees, feet, and fhoulders much fwelled, very red and inflamed, hot to the touch, and extremely painful, and he was totally incapable of moving himfelf. The fkin felt very hot, was rather moift, and emitted that acid effluvia fo peculiar to this difeafe, and which from long experience having found to be a conftant attendant, I confider as a characteriftic fymptom. His tongue was flightly furred, white, and rather moift; he complained of a confiderable degree of thirft, and could not get any fleep. The urine was exceedingly high coloured, and depofited a copious pink fediment, an appearance I have invariably obferved in this difeafe. The pulfe was full, hard, and beat 112 in a minute. I ordered him ten drops of the Tinct. Digitalis every fix hours, and a ftrict antiphlogiftic regimen.

My profeffional engagements prevented me from feeing him every day, as he lived fome diftance from me; it was therefore on the 17th I found that the pain, fwelling, and inflammation of the joints had in no wife diminifhed, and the tongue and the fkin were in the fame ftate as before. Indeed, the thirft was in fome degree leffened, the urine not fo high coloured and turbid, turbid, and the pulfe reduced to 104. Not having had any fleep, a draught with tinct. opii, gt. xxx. was ordered at night, and the dofe of tinct. digitalis increafed to fifteen drops.

I was much pleafed on the 19th to find the pain, inflammation, and fwelling in both knees fo much abated, as to enable him to move them freely. The feet and one hand were much better; the other hand and both fhoulders in much the fame ftate as before, as was alfo the tongue and urine; the fkin moift and much cooler, and the pulfe 80. The opiate had procured him a good night, which was repeated, and the tinct. digitalis continued. Not having had ftools, a draught with infus. fennæ was ordered.

The pain, inflammation, and fwelling, on the 21ft, was confined to the right arm, all the other parts being entirely free; the heat much diminifhed, the fkin moift, the urine the fame, and the pulfe 80. He complained of a flight head-ach, which however lafted but a fhort time. The opening draught had produced two ftools, but, not having flept, the tinct. opii was increafed to forty drops, the tinct. digitalis continued.

On the 23d, the rednefs and fwelling had entirely difappeared, though a confiderable degree of pain was felt in the knees and fhoulders, accompanied with much weaknefs. The fkin was cool, and both that and the tongue moift; the urine much paler, and without the copious pink-coloured fediment noticed in the beginning. The pulfe 84. He had not had much fleep, and no motion; the aperient draught was therefore repeated, the opiate omitted till that had operated, and the tinct. digit. continued as before. I found he had not been fo punctual in taking his medicine for the laft two days as he ought. He paffed the 24th and 25th much better; but having again omitted his medicine, I found on the 26th that he had a return of pain, fwelling, and inflammation in the right hand and foot. The pulfe was at 84, and the fkin cool and moift.

By the 28th, when I again faw him, the fwelling was almoft entirely fubfided, without the leaft appearance of inflammation or fixed pain; the little pain he *did* feel was flight and wandering. The skin continued cool and moift, the urine was much paler, the bowels open, the appetite returning, and he had comfortable fleep. I now ordered him mutton broth, calves-foot jelly, and fago, and reduced the tinct. digit. to three times in a day.

On vifiting him March 1, I found to my furprize that the pain and fwelling had returned into one hand, and, on the 3d, that it had extended to the other. The next day (4th) the fymptoms were become confiderably aggravated, and on inquiry difcovered, that he had very imprudently, and contrary to my exprefs directions, drank feveral glaffes of mead. The tongue tongue, though moiſt, had become brown, the skin hotter, and the pulſe 92. He complained of head-ach and want of ſleep, and was ſo ſenſible of his imprudence in deviating from my directions, that he was determined to follow them implicitly in future. I increaſed the doſe of tinct. digit. to twenty drops.

The pain and ſwelling on the 7th were leſs, and every ſymptom favourable; the pulſe 96. The tinct. digit. was increaſed to twenty-five drops.

On the 10th, I was happy to find every veſtige of inflammation entirely vaniſhed; he had neither pain nor ſwelling any where; his appetite was good, the pulſe 88, and the urine now depoſited a copious lateritious ſediment. From this time, none of his complaints returned; the drops were reduced to ten, three times in a day; he ſoon recovered his former ſtate of health, and has maintained it to this day, except occaſional ſlight erratic pains, which rheumatic patients are for the moſt part ſubject to in cold weather.

I muſt here beg leave to direct your attention to the leading circumſtances of this Caſe. The ſymptoms, undoubtedly, indicated an high phlogiſtic diatheſis; and I am perfectly convinced, that had blood been drawn, its appearance would have fully confirmed my opinion: Yet was this diſeaſe, in general ſo obſtinate a one, checked in the ſhort ſpace of five days after the exhibition of the Digitalis; and for the next ſix, the progreſs towards health was conſiderable. And it cannot eſcape your obſervation, that on the medicine being omitted, the ſymptoms increaſed; but by afterwards duly perſiſting in its uſe, the diſeaſe was finally eradicated, in little more than three weeks from the time I firſt ſaw him; and this was effected without one bleeding, or the exhibition of a ſingle grain of nitre or antimony. I cann[illegible] conclude this caſe without obſerving, that it was as ſevere a one at its commencement as any I ever ſaw.

My patient informed me, that ſome years ago he was confined in the ſame complaint to his bed, *ſeventeen weeks*, during which ſpace he was bled *nine or ten times*.

It may not be improper to add, that the tincture I uſed was in the proportion of an ounce of the leaves recently dried and powdered, to five of ſpt. of wine, and was prepared under my own inſpection, with the moſt accurate attention. The leaves were gathered in the month of Auguſt, from vigorous healthy plants five or ſix feet high, growing on an elevated ſituation, and in a light ſandy ſoil.

If you ſhould conſider the publication of this Caſe in your Journal as likely to be of any advantage to the medical ſcience, it is much at your ſervice. I am, &c.

*Ewell, Surrey, Jan. 8th, 1802.* EDM. PITTS GAPPER.

